# Barrack Schools as Fever Hospitals!

**Published:** 1895-02-23

**Authors:** 


### BARRACK SCHOOLS AS FEVER HOSPITALS!

A letter in the *British Medical Journal* points out that while, on the one hand, the Metropolitan Asylums Board is about to spend a large sum of money upon new hospitals, the Poor Law authorities, however anxious they may be to encourage the boarding out of pauper children, are hampered and tied down by the possession of vast barrack schools, which have been erected at great expense and must be used somehow, and the notable suggestion is propounded that the Asylums Board might take these buildings and use them as hospitals for infectious diseases! The suggestion only wants putting into form to stand condemned. Of all silly ways of frittering away money, probably the most wasteful is the attempt to make a hospital out of a building originally constructed for another purpose. The building of hospitals has become a special art, and the days are long gone by when the possession of "steam laundries, kitchen appliances, and dormitory furniture" could be looked on as making a building suitable for hospital purposes.

